# Influence of obesity and cardiometabolic makers on lipoprotein-associated phospholipase A_2_ (Lp-PLA_2_) activity in adolescents: the healthy young cross-sectional study

**DOI:** 10.1186/1476-511X-12-19

**Published:** 2013-02-15

**Authors:** Isis T da Silva, Anelise de Souza Timm, Nágila RT Damasceno

**Affiliations:** 1 Department of Nutrition, School of Public Health, University of Sao Paulo, Sao Paulo, SP, Brazil; 2 Faculdade de Saúde Pública, Departamento de Nutrição, Av Dr Arnaldo, 715, 01246-904, São Paulo, SP, Brazil

**Keywords:** Adolescent, Lp-PLA_2_, Cardiovascular risk, Obesity

## Abstract

**Background:**

Lipoprotein-associated phospholipase A_2_ activity (Lp-PLA_2_) is a good marker of cardiovascular risk in adults. It is strongly associated with stroke and many others cardiovascular events. Despite this, the impact of obesity on this enzyme activity and its relation to biomarkers of cardiovascular disease in adolescents is not very well investigated. The purpose of this article is to evaluate the influence of obesity and cardiometabolic markers on Lp-PLA_2_ activity in adolescents.

**Results:**

This cross-sectional study included 242 adolescents (10–19 years) of both gender. These subjects were classified in Healthy Weight (n = 77), Overweight (n = 82) and Obese (n = 83) groups. Lipid profile, glucose, insulin, HDL size, LDL(−) and anti-LDL(−) antibodies were analyzed. The Lp-PLA_2_ activity was determined by a colorimetric commercial kit. Body mass index (BMI), waist circumference and body composition were monitored. Food intake was evaluated using three 24-hour diet recalls. The Lp-PLA_2_ activity changed in function to high BMI, waist circumference and fat mass percentage. It was also positively associated with HOMA-IR, glucose, insulin and almost all variables of lipid profile. Furthermore, it was negatively related to Apo AI (β = −0.137; *P* = 0.038) and strongly positively associated with Apo B (β = 0.293; *P* < 0.001) and with Apo B/Apo AI ratio (β = 0.343; *P* < 0.001). The better predictor model for enzyme activity, on multivariate analysis, included Apo B/Apo AI (β = 0.327; *P* < 0.001), HDL size (β = −0.326; *P* < 0.001), WC (β = 0.171; *P* = 0.006) and glucose (β = 0.119; *P* = 0.038). Logistic regression analysis demonstrated that changes in Apo B/Apo AI ratio were associated with a 73.5 times higher risk to elevated Lp-PLA_2_ activity.

**Conclusions:**

Lp-PLA_2_ changes in function of obesity, and that it shows important associations with markers of cardiovascular risk, in particular with waist circumference, glucose, HDL size and Apo B/Apo AI ratio. These results suggest that Lp-PLA_2_ activity can be a cardiovascular biomarker in adolescence.

## Background

Obesity is developing very fast in all age groups, but the growth rate has been much more acute in children and adolescents [1]. According to the National Center for Health Statistics (NCHS), obesity increased from 5% to 18.1% among adolescents between 1976–1980 and 2007–2008 [1]. This profile is consistent with the Brazilian Institute of Geographic and Statistic, which showed that overweight in Brazilian adolescents increased from 16.7% (2002–2003) to 21.5% (2008–2009) [2].

The metabolic imbalance in obesity supports inflammation, insulin resistance and oxidation [3-5]. In adolescents, these components favor the early occurrence of atherosclerosis [6] by the emergence of chronic diseases like diabetes and abnormal lipid levels [7,8].

Previously Celik et al. (2009) proposed that the incidence of obesity in adolescence may perhaps to represent a first step to atherosclerosis in adults [9]. Many factors can be associated with cardiovascular diseases, and it is known that Lipoprotein-associated phospholipase A_2_ (Lp-PLA_2_) is a possible emerging biomarker [10]. Lp-PLA_2_ is an enzyme produced by inflammatory cells [11], that circulates on plasma linked in high proportion with low density lipoprotein (LDL) (85%) and less proportion to high density lipoprotein (HDL) (15%) [12]. Afterward, it was observed that Lp-PLA_2_ has the ability to hydrolysis oxidized phospholipids, reducing their biological activity [13]. However, this event results on the formation of lysophosphatidylcoline and isoprotanes, inflammatory components that participate in the development of atherosclerotic plaque [14].

Furthermore, it has been observed that Lp-PLA_2_, in adults, has association with other cardiovascular risk markers, such as LDL-C and C-reactive protein [15,16]. A recent review showed that Lp-PLA_2_ is related to cardiovascular events in adults, even after adjustment by Framingham risk score and C protein reactive [17]. Despite these results, the monitoring of Lp-PLA_2_ in children and adolescents is sparkly described in literature. In addition, it is not know if obesity is associated to change of Lp-PLA_2_ in adolescents. In this context, the aim of this article is to evaluate the influence of obesity and cardiometabolic markers on Lp-PLA_2_ activity in adolescents.

## Methods

### Study subjects

In a cross-sectional study, we evaluated boys and girls adolescents between ten and nineteen years old [18], selected from public schools (Sao Paulo, SP, Brazil). From five public schools, 2746 adolescents were enrolled in this study. This population was initially invited to participate in the nutritional screening (body mass index – BMI, weight/height^2^). After a preliminary nutritional status classification, all adolescents with overweight (n = 481), obesity (n = 135) and a randomized sub-sample of healthy weight (n = 756) adolescents were invited to the second phase of the study. A total of 261 adolescents completed all stage of data collection. 

The exclusion criteria were smoking, the use of alcohol (≥ 30.0 mL of ethanol/day for boys and ≥ 15.0 mL of etanol/day for girls) [19], the use of lipid-lowering drugs, the presence of any acute disease, the participation in other protocols research and pregnancy or breastfeeding. After an examination of collected data, 19 subjects were excluded due to the use of lipid-lowering and anti-inflammatory drugs during the collection.

The adolescents included in this study were distributed into 3 groups: healthy weight (HW), overweight (OV), and obese (OB), according to the classification of BMI, proposed by Cole et al. [20,21] for sex and age. The protocol study was approved by the Ethics Committee (School of Public Health, University of Sao Paulo) and followed the recommendations of the National Council for Health on Ethics in Research with Humans. All the parents of the adolescents signed an informed written consent.

### Food Intake

The food intake was evaluated by three 24-hour diet recalls, collected on non consecutive days, including one weekend day. The first recall was conducted by a direct interview and the others by phone interview. Energy (kcal), carbohydrates (g), lipids (g), proteins (g), saturated fat acid (g), monounsaturated fat acid (g), polyunsaturated fat acid (g), oleic acid (g), linoleic acid (g), linolenic acid (g) and fiber (g) were measured through the software *NutWin*® [22]. These data were adjusted by energy [23,24] and by intra-personal variability [25].

### Clinical and biochemical assessments

Height and weight were determined by a stadiometer (AlturaExata, TBW Brazil, Sao Paulo, SP, Brazil) and by a digital scale (Control, Plenna, Sao Paulo, SP, Brazil), respectively. Waist circumference (WC) was measured using a 1 mm precision flexible and inelastic tape (TBW Brazil®, Sao Paulo, SP, Brazil). Body composition was evaluated by bioelectric bioympedance technique, using the instrument tetrapolar Biodynamics®, model 450 (TBW, São Paulo, Brazil). Sexual maturation was evaluated according to Marshall and Tanner [26] and Marshall [27]. The levels of total cholesterol (TC), HDL-cholesterol (HDL-C), and triglycerides (TG) were determined by standard methods (Labtest Diagnóstica, MG, Brazil). The LDL-cholesterol (LDL-C) levels were calculated by the Friedewald equation [28]. Similarly, TC/HDL-C, LDL-C/HDL-C, no-HDL-C and TG/HDL-C ratios were calculated. The apolipoproteins AI (Apo AI) and B (Apo B) were evaluated by commercial kits (RANDOX®, Co, Antrim, United Kingdom). The concentrations of HDL-C and LDL-C were normalized by Apo AI and B levels, respectively. Posteriorly, the HDL size was measured by Laser-Light-Scattering method established by Lima and Maranhão [29].

The glucose was determined by an enzymatic and colorimetric commercial kit (Glicose PAP Liquiform® - Labtest, MG, Brazil). For insulin levels, the radioimmunoassay was applied using the standard method (Human Insulin-Specific RIA Kit^,^ Linco Research, St Charles, MO, USA). Following up, the insulin resistance was estimated by HOMA-IR index (homeostasis model assessment – insulin resistance), where: HOMA-IR = [fasting insulin level (μU/mL) × fasting glucose (mmol/L)] / 22.5 [30].

The electronegative low density lipoprotein LDL(−) was assessed by ELISA using monoclonal antibodies (MAb 1A3 and MAb 2C7), according to Damasceno *et al.*[31], while the autoantibodies against LDL(−) were evaluated by ELISA, according to Damasceno *et al.*[32].

Lp-PLA_*2*_ activity in plasma was determined by an enzymatic PAF-Acetylhydrolase Assay kit (Cayman Chemical Company, USA). The analyses were performed in duplicate, with its results expressed in nmol/min/mL.

### Statistical analysis

The statistical analyses were performed using the SPSS software, version 15.0. Following the evaluation of the data distribution by Kolmogorov-Smirnov test (*P* > 0.05), differences between groups were determined by ANOVA test (normally data) or Kruskal-Wallis and Mann–Whitney *U*-test (not normally distributed data). The *χ*^2^ test was used for comparison of categorical variables and results were expressed as relative frequencies (%). Initially, there were performed sexual maturation- and sex-specific analysis; nonetheless, the groups showed similar profiles. Therefore, these variables were not accepted as confounders. Univariated regression models were applied in order to determine the effect of anthropometric/body composition and lipid measures (as explanatory variables) on Lp-PLA_2_ activity (as dependent outcome). In order to explore the ability of the variables that showed association with Lp-PLA_2_, additive multivariated regression models were constructed using the following variables: BMI, WC,% fat mass, TC, LDL-C, TC/HDL-C, non-HDL-C, Apo AI, Apo B, Apo B/Apo AI, HDL size, insulin, glucose and HOMA. Results from regression models are showed as b-coefficients, R-squared values and *P*- value.

We have also performed the Odd Ratio (OR) using logistic regression. Regarding that there is no reference values previously established for Lp-PLA_2_, we proposed the highest quartile (Q4) of Lp-PLA_2_ activity (>15.9 nmol/min/mL) such as cutoff point. Statistical significance was established for *P*-value <0.05.

## Results

Table 1 shows the characteristics of adolescents included in the study, according to BMI. The proportion of girls (HW = 51%, OV = 45% and OB = 52%) were statistically similar between groups (*P* = 0.321). While the overweight adolescents (13.3 ± 1.9 years) was younger than both obese and healthy weight adolescents (*P* = 0.006), the OB group was marked by high fat mass percentage (32.2 ± 6.9%) and elevated central obesity (98 ± 16.5 cm), confirming previous BMI classification. It is worth to emphasize that food intake was similar between the groups.

**Table 1:** Characteristic of adolescents and food intake, according to BMI

	**HW (n = 77)**	**OV (n = 82)**	**OB (n = 83)**	***P***
**Boys, n (%)**	26 (35.7)	37 (52.6)	31 (44.4)	0.320
**Girls, n (%)**	51 (64.3)	45 (47.4)	52 (55.6)	
**Age, year**	14.4 (2.2)	13.3 (1.9)^*^	14.2 (2.6)^**^	0.006
**Sexual Maturation**				0.710
Pre-pubertal	4 (5)	7 (9)	6 (7)	
Pubertal	73 (95)	75 (92)	77 (93)	
**BMI, kg/m**^**2**^	20.3 (2.1)	25.0 (1.9)^*^	32.4 (5.9)^*.**^	<0.001
**WC, cm**	69 (5.8)	80.7 (5.7)^*^	98 (16.5)^*.**^	<0.001
**Fat mass,%**	19.4 (7.9)	24.5 (6.6)^*^	32.2 (6.9)^*.**^	<0.001
**Lean mass,%**	76.1 (13.9)	71.3 (9.8)^*^	67.0 (7.5)^*.**^	<0.001
**Food intake**				
Energy, kcal	1522 (471)	1446 (438)	1563 (521)	0.620
Carbohydrates, g	211 (20)	212 (19)	207 (27)	0.706
Proteins, g	64 (11)	64 (10)	66 (15)	0.719
Fat, g	50 (8)	51 (6)	51 (7)	0.313
SFA, g	17.7 (3.1)	17.4 (2.8)	17.7 (3.3)	0.480
PUFA, g	8.5 (2.0)	9.2 (2.0)	9.0 (2.7)	0.213
MUFA, g	17.3 (2.8)	17.9 (2.8)	17.7 (3.3)	0.159
Oleic acid, g	9.2 (2.2)	9.9 (1.7)	10.7 (2.8)	0.050
Linoleic acid, g	3.9 (1.0)	4.2 (1.0)	4.0 (1.0)	0.142
Linolenic acid, g	0.4 (0.1)	0.5 (0.1)	0.4 (0.1)	0.079
Fiber, g	11.1 (2.8)	10.7 (2.3)	10.7 (2.8)	0.948

HW, healthy weight; OV, overweight; OB, obese; BMI, body mass index; WC, waist circumference; SFA, saturated fat acid; PUFA, polyunsaturated fat acid; MUFA, monounsaturated fat acid.

Continuous variables were described as mean (standard deviation).

Categorical variables were described as sample (n) and percentage (%).

*P < 0.05 *vs* HW group. **P < 0.05 *vs* OV group.

As to the lipid profile, HDL-C was lower in OV and OB groups in comparison to HW group (*P* = 0.027; *P* < 0.001, respectively), and TG was higher in OB group (*P* = 0.026). The TC/HDL-C and TG/HDL-C ratios confirmed this tendency, where obese adolescents showed higher values in relation to healthy weight adolescents (*P* = 0.015). Regarding the apolipoproteins, they were similar between groups, except for Apo AI and Apo B/Apo AI ratio, where the OB group showed lower (*P* = 0.001) and higher (*P* = 0.002) levels, respectively, than HW group (Table 2). The insulin levels and HOMA-IR were higher in OV (*P* = 0.002 and *P* = 0.011, respectively) and OB groups (*P* < 0.001; *P* < 0.001, respectively) than in HW group. The similar pattern was observed for LDL(−), where obese and overweight adolescents showed values statistically higher than HW group (*P* = 0.049; *P* = 0.017, respectively). An opposite profile was noted for anti-LDL(−) (*P* = 0.045; *P* = 0.010, respectively) (Table 2).

**Table 2:** Adolescent’s biochemical profile, according to BMI

	**HW (n = 77)**	**OV (n = 82)**	**OB (n = 83)**	***P***
**TC, mg/dL**	143 (32.1)	140 (34.3)	142 (39.8)	0.864
**LDL-C, mg/dL**	86 (33.5)	85 (35.6)	89 (40.1)	0.800
**HDL-C, mg/dL**	42 (13.5)	37 (13.3)^*^	35 (12.6)^*^	<0.001
**TG, mg/dL**	75 (29.7)	87 (49.7)	91 (45.1)^*^	0.07
**TG/HDL-C**	1.90 (0.86)	2.69 (1.98)^*^	3.07 (2.24)^*^	0.001
**TC/HDL-C**	3.67 (1.39)	4.02 (1.72)	4.59 (2.16)^*^	0.021
**LDL-C/HDL-C**	1.81 (0.52)	1.90 (0.90)	1.79 (0.71)	0.051
**Non HDL-C**	100.9 (33.9)	102.6 (38.3)	107.1 (43.2)	0,575
**Apo AI, mg/dL**	120.9 (18.0)	113.7 (19.6)	109.1 (21.2)^*^	0.001
**Apo B, mg/dL**	62.8 (10.6)	65.2 (15.8)	65.2 (17.5)	0.533
**Apo B/Apo AI**	0.53 (0.12)	0.60 (0.15)	0.61 (0.16)^*^	0.003
**LDL-C/ApoB**	1.38 (0.4)	1.33 (0.48)	1.37 (0.53)	0.810
**HDL-C/ApoA**	0.35 (0.09)	0.33 (0.12)	0.32 (0.10)	0.122
**LDL (−), mg/mL**	8.9 (5)	10.8 (4.9)	10.5 (5.2) ^*^	0.042
**Anti LDL(−), mg/mL**	8.5 (2.6)	7.5 (2.7)^*^	7.2 (3.0)^*^	0.010
**HDL size, nm**	10.8 (2.7)	10.8 (2.3)	10.9 (3.2)	0.638
**Glucose, mg/dL**	79.3 (12.8)	78.0 (12.5)	82.0 (13.2)	0.130
**Insulin, uU/mL**	15.3 (7.1)	19.8 (8.2)^*^	25.9 (14.5)^*;**^	<0.001
**HOMA-IR**	3.0 (1.5)	3.9 (2.0)^*^	5.3 (3.1)^*;**^	<0.001

HW, healthy weight; OV, overweight; OB, obese; BMI, Body Mass Index; WC, waist circumference, HOMA-IR, homeostasis model assessment of insulin resistance; TC, total cholesterol; LDL, low density lipoprotein; HDL, high density lipoprotein.

Results were described as mean (standard deviation). *P < 0.05 *vs* HW group. **P < 0.05 *vs* OV group.

**Figure 1:**
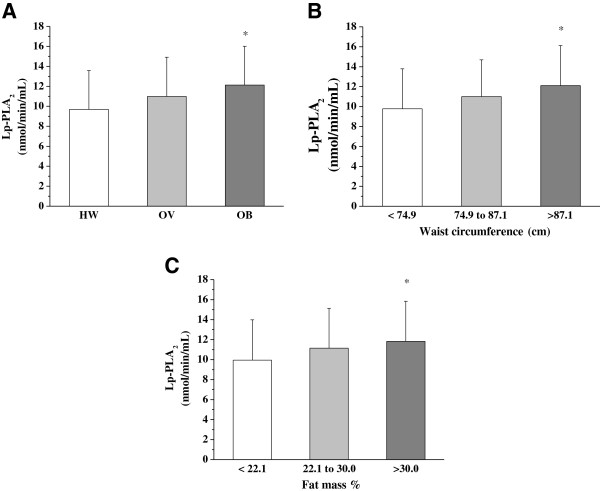
**Lp-PLA**_**2**_**activity in adolescents according to BMI, tertiles of waist circumference and fat mass percentage.A**: Lp-PLA_2_ activity stratified by BMI. **B**: Lp-PLA_2_ activity stratified by waist circumference tertiles. **C**: Lp-PLA_2_ activity stratified by fat mass percentage tertiles. *P = 0.001 *vs* HW group or *vs* Lower tertile. HW, healthy weight; OV, overweight; OB, obese; BMI, Body Mass Index; WC, waist circumference.

The Figure 1 shows that Lp-PLA_2_ activity changed in function to obesity. The negative impact of obesity on Lp-PLA_2_ activity was reinforced by tertiles of waist circumference and fat mass percentage. Regarding this profile, correlations between Lp-PLA_2_ and % fat mass, WC and BMI in all groups were analyzed, but it weren’t statistically significant, excepted for % fat mass that was negatively related to Lp-PLA_2_ in the OV group (r = −0.238 p = 0.033).

Table 3 shows the association between Lp-PLA_2_ activity and cardiometabolic parameters. Lp-PLA_2_ activity was positively associated with almost all variables of lipid profile, except HDL-C and TG with which there was not correlation. Besides, Lp-PLA_2_ was negatively related to Apo AI (β = −0.137; *P* = 0.038) and strongly positively associated with Apo B (β = 0.293; *P* < 0.001) and with Apo B/Apo AI ratio (β = 0.343; *P* < 0.001). Other evidences are the positive associations with insulin, HOMA-IR and glucose. The enzyme activity was also positively associated with BMI (β = 0.195; *P* = 0.003), WC (β = 0.270; *P* < 0.001), fat mass percentage (β = 0.186; *P* = 0.004). These significant associations explained 64.5% of variability of Lp-PLA_2,_ while the non significant represented 4.1%.

**Table 3:** **Univariated regression model between Lp-PLA**_**2**_**and cardiometabolic factors**

	**Lp-PLA**_**2**_		
	**R**^**2**^	P*	**β**
**BMI, kg/m**^**2**^	0.038	0.003	0.195
**WC, cm**	0.073	0.000	0.270
**Fat mass,%**	0.034	0.004	0.186
**TC, mg/dL**	0.035	0.004	0.186
**LDL-C, mg/dL**	0.016	0.051	0.128
**HDL-C, mg/dL**	0.001	0.671	−0.028
**TG, mg/dL**	0.008	0.163	0.091
**TG/HDL-C**	0.007	0.199	0.084
**LDL-C/HDL-C**	0.010	0.121	0.121
**TC/HDL-C**	0.018	0.042	0.133
**Non HDL-C**	0.022	0.022	0.149
**HDL-C/ApoAI**	0.001	0.585	0.036
**Apo AI, mg/dL**	0.019	0.038	−0.137
**Apo B, mg/dL**	0.082	0.000	0.293
**Apo B/Apo AI**	0.118	0.000	0.343
**LDL-C/ApoB**	0.000	0.775	−0.019
**LDL(−), mg/mL**	0.001	0.706	0.025
**Anti LDL(−), mg/mL**	0.013	0.078	−0.115
**HDL size , nm**	0.085	0.000	−0.291
**Insulin, uU/mL**	0.039	0.002	0.198
**Glucose, mg/dL**	0.019	0.034	0.138
**HOMA-IR**	0.047	0.001	0.216

BMI, Body Mass Index; WC, waist circumference, HOMA-IR, homeostasis model assessment of insulin resistance; TC, total cholesterol; TG, triglycerides; LDL, low density lipoprotein; HDL, high density lipoprotein.

Variables in log form, except for percentages. *Significant level (*P* < 0.05).

Given the multiple associations obtained in the univariated test, we analyzed the better model of multivariable association in order to understand the Lp-PLA_2_ activity in adolescents. The better predictor model was featured by Apo B/Apo AI (β = 0.327; *P* < 0.001)

HDL size (β = −0.326; *P* < 0.001), WC (β = 0.171; *P* = 0.006) and glucose (β = 0.119; *P* = 0.038) (Table 4). This model explained 26.3% of variability of Lp-PLA_2_.

**Table 4:** **Multivariable linear regressions between Lp-PLA**_**2**_**and cardiometabolic factors**

	**Lp-PLA**_**2**_		
	**R**^**2**^	P*	**β**
***Model***	0.263		
**Apo B/Apo AI**		0.000	0.327
**HDL size**		0.000	−0.326
**WC**		0.006	0.171
**Glucose**		0.038	0.119

WC, waist circumference; HDL size; high density lipoprotein size.

Variables in log form.

*Significant level (*P* < 0.05).

Using logistic regression analysis, we were able to determine the impact of cardiovascular risk factors on Lp-PLA_2_ activity (Table 5). Compared to adolescents with lower Lp-PLA_2_ activity (<15.9), those with higher values (≥15.9) exhibited significant association with TC (OR 1.012, 95% CI = 1.004-1.020, *P* = 0.004), LDL-C (OR 1.012, 95% CI = 1.004-1.020, *P* = 0.003), HOMA-IR (OR 1.127, 95% CI = 1.010-1.258, *P* = 0.032), Apo B (OR 1.042, 95% CI = 1.020-1.064, *P* < 0.001), Apo B/Apo AI (OR 73.53, 95% CI = 8.627-626.665, *P* < 0.001). The change in Apo B/Apo AI ratio was related to a 73.5 times higher risk to have a high value of Lp-PLA_2_ (≥15.9).

**Table 5:** **Odd Ratio for Lp-PLA**_**2**_**activity highest quartile**

	**Lp-PLA**_**2**_		
	**<15.9**	**≥15.9**	P*
**TC, mg/dL**	1 ^Ref^	1.012	0.004
**LDL-C, mg/dL**	1 ^Ref^	1.012	0.003
**HDL-C, mg/dL**	1 ^Ref^	0.983	0.162
**TG, mg/dL**	1 ^Ref^	1.004	0.178
**HOMA-IR**	1 ^Ref^	1.127	0.032
**HDL size, nm**	1 ^Ref^	0.930	0.245
**LDL(−), mg/mL**	1 ^Ref^	1.000	0.757
**Anti LDL(−), mg/mL**	1 ^Ref^	0.878	0.020
**Apo AI mg/dL**	1 ^Ref^	0.993	0.374
**Apo B mg/dL**	1 ^Ref^	1.042	0.000
**Apo B/Apo AI**	1 ^Ref^	73.5	0.000
**LDL-C/ApoB**	1 ^Ref^	1.265	0.443
**HDL-C/ApoAI**	1 ^Ref^	0.123	0.168
**BMI, kg/m**^**2**^	1 ^Ref^	1.022	0.340
**WC, cm**	1 ^Ref^	1.014	0.126
**Fat mass,%**	1 ^Ref^	1.015	0.379

HW, healthy weight; OV, overweight; OB, obese; BMI, Body Mass Index; WC, waist circumference, HOMA-IR, homeostasis model assessment of insulin resistance; TC, total cholesterol, TG, triglycerides; LDL, low density lipoprotein; HDL, high density lipoprotein.

*Significant level (*P* < 0.05).

## Discussion

This study showed that Lp-PLA_2_ activity in adolescence is associated with many cardimetabolic parameters. The factors that had an impact in the activity of the enzyme, from the most to the least important, were the Apo B/Apo AI ratio, waist circumference, glucose and HDL size.

Our results demonstrated the negative effect of obesity on Lp-PLA_2_ activity, which were reinforced by waist circumference and fat mass percentage. Among the anthropometric variables the waist circumference was the most important factor explaining the variation in the Lp-PLA_2_ activity. This fact confirms the relevance of the body fat location. Interestingly, analysis of correlations in individual groups did not show association with healthy weight and overweight adolescents, except for fat mass percentage and Lp-PLA_2_ activity. In this sense, Taylor and Hergenroeder demonstrated that elevated waist circumference in male adolescents was associated with an increased risk of cardiometabolic disease and overweight females with elevated waist circumference were likely to have elevated blood pressure [33].

In addition, we demonstrated for the first time the strong impact of Apo B/Apo AI ratio on changes in Lp-PLA_2_ activity; more specifically, the changes in these apolipoproteins were associated with a 73.5 times higher risk of elevated Lp-PLA_2_ activity. Recently, Hatoum *et al.*[34], studying patients between 50 and 60 years old, observed that the enzyme was modestly associated with total cholesterol, LDL-C, Apo B and BMI; however, the lipid adjustment attenuates the relation between BMI and Lp-PLA_2_. The atherogenic role of Lp-PLA_2_ was widely associated to LDL-C [16,35]. On the contrary, Okamura *et al.*[16] suggested that Lp-PLA_2_ in HDL plays an antiaterogenic action. This observation was reinforced by a high LDL-Lp-PLA_2_ to HDL-Lp-PLA_2_ ratio verified in patients with atrial fibrillation. In the same way, Rizos *et al.*[12] and Lagos *et al.*[36] observed a high total Lp-PLA_2_ activity in patients with metabolic syndrome, however, HDL-Lp-PLA_2_ showed low activity. Accordingly, our results confirm the impact of HDL and LDL on Lp-PLA_2_ activity given the strong association of this enzyme with Apo B/Apo AI ratio, that was the most important parameter related to the risk of increasing of Lp-PLA_2_ activity in adolescents.

The negative association of Lp-PLA_2_ with HDL-size signals a possible scenario where the functionality of HDL particles is impaired. Accordingly, Pascot *et al*., [37] showed that the small HDL particle has been associated with atherogenic dyslipidemic profile and hyperinsulinemia. More recently, Medina-Urrutia *et al.*[38] demonstrated that adolescents with small HDL have reduced HDL-C, high triglycerides and HOMA-IR. Rizos *et al.*[12] verified that patients with metabolic syndrome have higher Lp-PLA_2_ than the control group, but the enzyme linked to HDL-Lp-PLA_2_ was lower in this group and negatively associated with HOMA-IR.

Tsimikas *et al.*[39], analyzing patients with or without cardiovascular events, observed that Lp-PLA_2_ was positively correlated with HOMA-IR in the two groups; nonetheless, the correlation was higher in the cardiovascular event group. In this fashion, our study demonstrated that this enzyme was positively correlated with glucose, reinforcing the notion of the negative impact of obesity on glucose metabolism.

Previous studies have shown that Lp-PLA_2_ is a good marker for cardiovascular risk in adults [10,15,40]. For instance, the Lp-PLA_2_ Studies Collaboration showed that the enzyme activity was positively correlated with non-HDL-C, LDL-C, Apo B and negatively correlated with HDL-C and Apo AI [10]. In the same way, Sabatine *et al.*[41] emphasized that Lp-PLA_2_ is an important predictor of coronary revascularization and unstable angina, and can also be treated as a new risk factor.

In contrast to many investigations related to negative impact of obesity on lipids, few studies including children and adolescents that linked obesity, lipid profile and Lp-PLA_2_. At the moment, there are only four studies evaluating Lp-PLA_2_ in children and adolescents. First, Okada *et al.*[42] showed that the Lp-PLA_2_ concentration of 17 obese children (11.9 ± 0.7 years old) was positively correlated with weight, waist/height ratio, subscapular/triceps ratio and LDL-C level. Castro *et al.*[43] compared diabetic young adults and adolescents (24.9 ± 7.8 years old) with controls (24.3 ± 9.6 years old), observing higher Lp-PLA_2_ activity and elevated susceptibility for oxidized LDL in diabetic patients. Subsequently, Nagel *et al.*[44], studying children with 10 years old, observed that overweight was positively associated with cardiometabolic markers, including Lp-PLA_2_ concentration. In 2011, Motykova *et al*. [45], examining a group of non-diabetic obese/overweight children, verified that body weight reduction determined a drop of Lp-PLA_2_ levels, but even after this alteration the concentrations remain elevated. Our study confirms the negative impact of Lp-PLA_2_ and amplifies these results in function of sample size, number cardiometabolic markers and the age of the adolescents included.

In addition, many studies have shown that Lp-PLA_2_ activity is elevated in hypercholesterolemic, diabetic and metabolic syndrome patients [12,46]. Nambi *et al.*[47] associated C-reactive protein and Lp-PLA_2_ to traditional risk factors for cardiovascular disease and proposed that these variables can be especially useful in individuals that are in intermediate risk according to these evaluations. Hence, Lp-PLA_2_ appears to be an important link between oxidation, inflammation, and altered lipid profile and insulin resistance on cardiovascular diseases. This action maybe involves the formation of lysophosphatidylcholine and isoprostanes that are the bioactive lipids released by Lp-PLA_2_[14].

In spite of the important results obtained, we believe that this study shows limitations directly related to the design (cross-sectional) that limits the establishment of the causal impact of our results on prevalence of clinical events.

Finally, we conclude that in adolescence, Lp-PLA_2_ changes in function of obesity, and that it shows important associations with markers of cardiovascular risk, in particular with waist circumference, glucose, HDL size and Apo B/Apo AI ratio. This supports the hypothesis that Lp-PLA_2_ can be a biomarker of cardiovascular risk in adolescence, however for confirm this; new prospective cohorts will be required.

## Abbreviations

Anti-LDL(−): Autoantibodies against electronegative low density lipoprotein; Apo AI: Apolipoprotein AI; Apo B: Apolipoprotein B; BMI: Body mass index; HDL: High density lipoprotein; HOMA-IR: Homeostasis model assessment – insulin resistance; HW: Healthy weight; LDL(−): Electronegative low density lipoprotein; Lp-PLA_2_: Lipoprotein-associated phospholipase A_2_; OB: Obese; OR: Odd Ratio; OV: Overweight; TG: Triglycerides; WC: Waist circumference.

## Competing interests

The authors declare no competing interests.

## Authors’ contributions

ITS, AST and NRTD participated of the acquisition, analysis, and interpretation of the data. ITS wrote and organized the structure of the manuscript, figures and tables. NRTD critically reviewed and revised the manuscript. All authors approved the final version of the manuscript.
